# Role of *TRAK1* variants in epilepsy: genotype–phenotype analysis in a pediatric case of epilepsy with developmental disorder

**DOI:** 10.3389/fnmol.2024.1342371

**Published:** 2024-02-12

**Authors:** Ren-Ke Li, Yu-Rong Xiong, Shu-Jing Pan, Wen-Ting Lei, Xiao-Mei Shu, Xiao-Qi Shi, Mao-Qiang Tian

**Affiliations:** Department of Pediatrics, Affiliated Hospital of Zunyi Medical University, Children’s Hospital of Guizhou Province, Zunyi, China

**Keywords:** *TRAK1* gene, epilepsy, biallelic variant, genotype and phenotype, developmental disorder

## Abstract

**Purpose:**

The *TRAK1* gene is mapped to chromosome 3p22.1 and encodes trafficking protein kinesin binding 1. The aim of this study was to investigate the genotype–phenotype of *TRAK1*-associated epilepsy.

**Methods:**

Trio-based whole-exome sequencing was performed on a cohort of 98 patients with epilepsy of unknown etiologies. Protein modeling and the VarCards database were used to predict the damaging effects of the variants. Detailed neurological phenotypes of all patients with epilepsy having *TRAK1* variants were analyzed to assess the genotype–phenotype correlations.

**Results:**

A novel *TRAK1* compound heterozygous variant comprising variant c.835C > T, p.Arg279Cys and variant c.2560A > C, p.Lys854Gln was identified in one pediatric patient. Protein modeling and VarCards database analyses revealed that the variants were damaging. The patient received a diagnosis of early infantile epileptic spasms with a developmental disorder; he became seizure-free through valproate and adrenocorticotropic hormone treatment. Further results for six variants in 12 patients with epilepsy indicated that biallelic *TRAK1* variants (including homozygous or compound heterozygous variants) were associated with epilepsy with developmental disorders. Among these patients, eight (67%) had epileptic spasms and seven (58%) were intractable to anti-seizure medicines. Moreover, eight patients experienced refractory status epilepticus, of which seven (88%) died in early life. To our knowledge, this is the first reported case of epilepsy caused by *TRAK1* compound heterozygous variants.

**Conclusion:**

Biallelic *TRAK1* variants can cause epilepsy and developmental disorders. In these patients, seizures progress to status epilepticus, suggesting a high risk for poor outcomes and the requirement of early treatment.

## 1 Introduction

The *TRAK1* gene (OMIM* 608112), mapped to 3p22.1, encodes trafficking protein kinesin binding 1 (TRAK1) (Gilbert et al., 2006), which has 953 amino acids and contains a HAP1-N domain in the N-terminal and a kinesin-binding Milton domain in the C-terminal (Gilbert et al., 2006). *TRAK1* has a significant role in mitochondrial axonal transport and in the regulation of endocytic GABA-A receptor trafficking (Barel et al., 2017). *TRAK1* is highly expressed in the spinal cord and moderately expressed in all other tissues and specific brain regions (Kikuno et al., 1999). Mice homozygous for the knockout allele died prematurely.^1^ The gnomAD database revealed either no frequencies or low frequencies for *TRAK1* homozygous or heterozygous variants. In humans, *TRAK1* homozygous variants have been related to developmental and epileptic encephalopathy 68 (DEE68, OMIM# 618201) (Barel et al., 2017)—an autosomal recessive neurological disorder characterized by the onset of twitching and/or myoclonic jerks in infancy. The disease progresses to refractory generalized tonic-clonic seizures, which often contribute to status epilepticus and loss of developmental milestones. When seizures progress to status epilepticus, most patients die within several months to years. Other clinical features of developmental and epileptic encephalopathy 68 include delayed development, axial hypotonia, spasticity, seizures, and clonus; brain imaging may show cortical atrophy (Barel et al., 2017). *TRAK1* heterozygous variants have been reported with a wide spectrum of diseases, including developmental disorders, autism spectrum disorder, schizophrenia, neurodevelopmental disorders, neuralgia, and trigeminal and congenital diaphragmatic hernia (Xu et al., 2011; Iossifov et al., 2014; Deciphering Developmental, and Disorders, 2017; Turner et al., 2019; Bacchelli et al., 2020; Dong et al., 2020; Qiao et al., 2021). There are currently no medications available to mitigate the effects associated with the *TRAK1* variant; moreover, *TRAK1* compound heterozygous variants have not been reported.

In this study, we performed trio-based whole-exome sequencing (WES) on a cohort of 98 patients with epilepsy of unknown etiologies. We identified one patient with the *TRAK1* compound heterozygous variant who was characterized by early infantile epileptic spasms and developmental disorders. We assessed the correlation between *TRAK1* variants and epilepsy and further analyzed the genotype–phenotype relationships in *TRAK1*-associated epilepsy based on this and previous cases.

## 2 Materials and methods

### 2.1 Subjects

We recruited 98 patients (58 male and 40 female) with epilepsy of unexplained etiologies from the Affiliated Hospital of Zunyi Medical University, from January 2019 to October 2023. The age of the recruited patients ranged from 1 month to 14 years. Clinical information on the affected patients, including sex, age of onset, type and frequency of seizures, general and neurological examination results, family history, and response to anti-seizure medicines (ASMs), was collected through a face-to-face review conducted by the authors. Magnetic resonance imaging (MRI) scans were performed to detect structural abnormalities in the brain. Long-term video electroencephalography (EEG) monitoring was performed, including hyperventilation, intermittent photic stimulation, open–close eye tests, and sleep recordings. Epileptic seizures and epilepsy syndromes were diagnosed according to the criteria of the Commission on the Classification and Terminology of the ILAE (1981, 1989, 2001, 2010, 2017, and 2022) (Engel, 2006; Scheffer et al., 2017; Specchio et al., 2022).

This study adhered to the guidelines of the International Committee of Medical Journal Editors on patient consent for research or participation and was approved by the Ethics Committee of the Affiliated Hospital of Zunyi Medical University (approval number: KLLY-2021-026). Written informed consent was provided by the patients’ legal guardians.

### 2.2 Trio-based whole-exome sequencing

Blood samples were obtained from the probands and their parents to determine the origins of the genetic variants. Genomic DNA was extracted from peripheral blood using a QuickGene DNA Whole Blood Kit (Fujifilm, Tokyo, Japan). Trio-based WES was performed using an Illumina HiSeq 2500/4000 platform (MyGenostics, Beijing, China). A case-by-case analytical method was used to identify causative variants. We prioritized rare variants with a minor allele frequency < 0.005 in the gnomAD database.^2^ Potentially pathogenic variants, including frameshift, nonsense, canonical splice site, initiation codon, and missense variants were predicted to be damaging using *in silico* tools. We screened for potential disease-causing variants in each family were sifted using five models: (1) epilepsy-associated gene model, (2) *de novo* dominant model, (3) autosomal recessive inheritance model (including homozygous or compound heterozygous variants), (4) X-linked model, and (5) co-segregated model. Novel epilepsy genes were characterized by destructive, *de novo*, biallelic, and hemizygous variants and confirmed using Sanger sequencing. *TRAK1* emerged as a candidate gene for compound heterozygous variants in this cohort. The *TRAK1* variants were annotated based on NM_001042646 and confirmed by Sanger sequencing.

### 2.3 Molecular structure analysis

To assess the effect of the *TRAK1* variant on protein structure, protein modeling was performed by using the AlphaFold Protein Structure Database (Jumper et al., 2021). Swiss-PdbViewer^3^ was used to analyze the three-dimensional protein structures of the TRAK1 protein model.

### 2.4 Protein stability

Grantham scores (Grantham, 1974) and multiple common tools from the VarCards database^4^, including SIFT, FATHMM_MKL, and fitCons, were used to predict the damaging effects of the variants. Higher Grantham and fitCons scores corresponded to more deleterious effects, and SIFT scores ≤ 0.5 and FATHMM_MKL scores ≥ 0.5 indicated deleterious effects. The I-Mutant 2.0 program^5^ was used to predict the effect of missense variants on protein stability (Capriotti et al., 2005). Changes in protein stability were evaluated based on free energy change (DDG, kcal/mol), where DDG < 0 indicated decreasing protein stability and DDG > 0 indicated increasing protein stability.

### 2.5 Genotype–phenotype analysis of *TRAK1*-associated epilepsy

To evaluate the relationship between *TRAK1* variants and epilepsy, we conducted an exhaustive search for *TRAK1* variants associated with epilepsy on PubMed until September 2023. We identified studies published in English using the following keywords: “*TRAK1*,” “epilepsy,” “seizure,” and “DEE68.” All *TRAK1* pathogenic variants associated with epilepsy and detailed neurological phenotypes were analyzed.

## 3 Results

### 3.1 Case report

**FIGURE 1:**
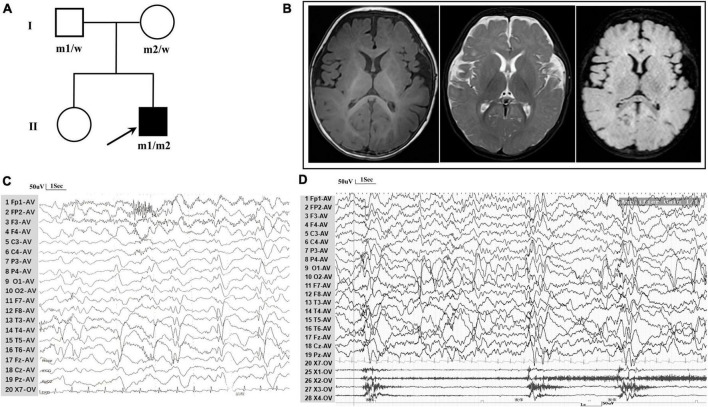
Clinical data for patient with *TRAK1* variant. **(A)** Pedigree of family. The filled arrow indicates the proband (m1 and m2 indicate mutant type and w indicates the wild type). **(B)** Brain MRI reveals Sylvian fissure dilation in T1-weighted imaging, T2-weighted imaging, and T2-FLAIR imaging. **(C)** Background EEG reveals bilateral posterior atypical hypsarrhythmia (obtained at the age of 7 months). **(D)** Interictal EEG reveals epileptic spasms (obtained at the age of 7 months).

The patient, a 7-month-old boy with a healthy sister, was the second child of a non-consanguineous Asian family (Figure 1A) and first visited our hospital in April 2023 because of an epileptic spasm. Since 5 months of age, he had often (two to three times daily) exhibited epileptic spasms in clusters either before or after waking. The frequency of seizures gradually increased by the age of 6.5 months. The longest seizure time was 1–2 min, and the interval between seizures was a few seconds. The patient was severely exhausted and could not sit independently after seizures. His vital signs were stable, and head, cardiopulmonary, and abdominal examinations were negative. No special conditions were observed during the nervous system examination. Birth, growth, development, and family history were normal. No obvious abnormalities were observed in routine blood tests, biochemistry, or other laboratory examinations. A brain MRI revealed Sylvian fissure dilation (Figure 1B). EEG revealed bilateral posterior atypical hypsarrhythmia and epileptic spasms in clusters (Figures 1C, D). A mild developmental delay was observed, and he was unable to walk independently at 1 year of age. Gesell Developmental Observation-Revised screening revealed a mild delay in gross motor development, language, and social-emotional responses. His seizures were not responsive to valproate (24 mg/kg/d). At the age of 7.5 months, he was administered adrenocorticotropic hormone (ACTH) (150 U/m^2^/d) treatment combined with valproate for a total of 14 days. Finally, he became seizure-free at the age of 8 months.

### 3.2 *TRAK1* variant identification

**FIGURE 2:**
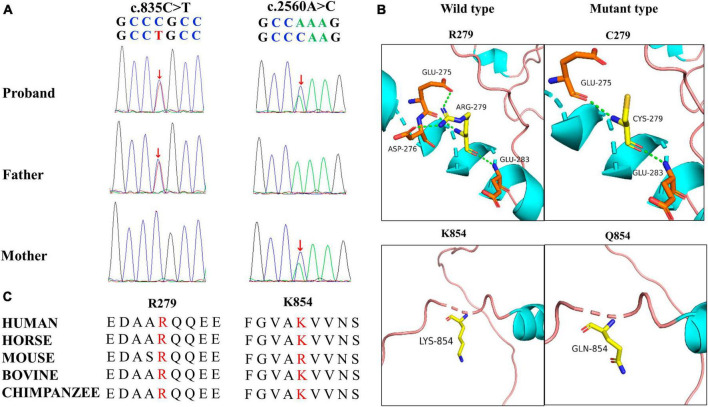
Genetic data for patient with *TRAK1* variant. **(A)** Variants c.835C > T (m1) and c.2560A > C (m2) were identified through whole-exome sequencing and confirmed by using Sanger sequencing. Arrows indicate the positions of the variants. All variants were inherited from the parents. **(B)** Hydrogen bond changes of the *TRAK1* variant. **(C)** Variant amino acids in our patient were conserved in multiple species.

A novel *TRAK1* compound heterozygous variant (NM_001042646: c.835C > T, p.Arg279Cys and c.2560A > C, p.Lys854Gln) was identified in the non-consanguineous Asian family (Figure 1A). The missense variant p.Arg279Cy in exon 8 was inherited from the father, and the missense variant p.Lys854Gln in exon 16 was inherited from the mother (Figure 2A). The two missense variants that presented at low or no allele frequencies in the gnomAD database (Table 1) were autosomal recessive and inherited from asymptomatic parents (Figure 1A).

**TABLE 1:** Genetic features of the individual for *TRAK1* variants identified in this study.

Position	cDNA change (NM_0010 42646)	Protein change	MAF	MAF-EAS	SIFT	FATHMM_MKL	fitCons	Grantham score
Chr 3: 42234632	c.835C > T	Arg279Cys	2.48 × 10^–5^	1.50 × 10^–4^	D (0.003)	D (0.944)	D (0.737)	180
Chr 3: 42264927	c.2560A > C	Lys854Gln	–	–	T (0.145)	D (0.948)	D (0.732)	53

Chr, chromosome; D, damaging; fitCons, fitness consequence; MAF, minor allele frequency from Genome Aggregation Database; MAF-EAS, minor allele frequency from East Asian population in Genome Aggregation Database; SIFT, sorting intolerant from tolerant; T, tolerable.

### 3.3 Structural alteration of the *TRAK1* protein

Swiss-PdbViewer was used to analyze the molecular effects of the missense variants. Among the novel *TRAK1* compound heterozygous variants, Arg279 formed three hydrogen bonds with residues Glu275, Asp276, and Glu283. The missense variant p.Arg279Cys decreased hydrogen bonding with residue Asp276; Lys854 did not form hydrogen bonds with any residue; and neither lysine nor glutamine at residue 854 formed hydrogen bonds with any residue (Figure 2B). Notably, the amino acid residues of the two missense variants were highly conserved among various species (Figure 2C). The two variants were predicted to be damaging based on at least three *in silico* tools and Grantham scoring (Table 1). I-Mutant 2.0 indicated that variant p.Arg279Cys had little effect on protein stability (DDG = 0.21 kcal/mol), whereas variant p.Lys854Gln would affect protein stability (DDG = −0.79 kcal/mol).

### 3.4 Role of *TRAK1* variants in epilepsy

**FIGURE 3:**
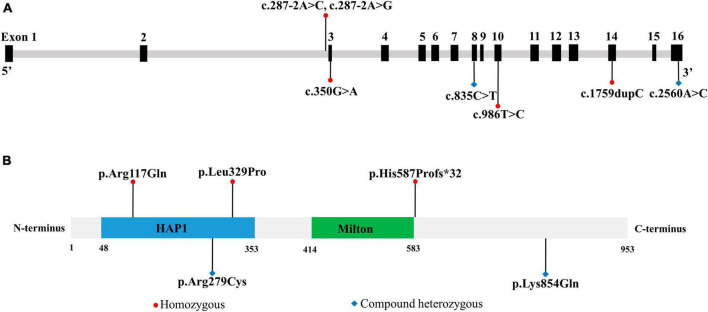
Location of *TRAK1* variant sites in patients with epilepsy. **(A)** Genomic location of *TRAK1* variants sites. **(B)** Schematic diagram of TRAK1 protein variants sites.

To evaluate the relationship between *TRAK1* variants and epilepsy, we summarized and analyzed relevant cases, including neurological phenotypes. We identified 13 *TRAK1* variants in 19 previously reported cases (Xu et al., 2011; Iossifov et al., 2014; Anazi et al., 2017; Barel et al., 2017; Deciphering Developmental, and Disorders, 2017; Sagie et al., 2018; Turner et al., 2019; Bacchelli et al., 2020; Dong et al., 2020; Mitani et al., 2021; Qiao et al., 2021). Of the thirteen variants, four were destructive variants (three frame shifts and one deletion), one was a gross duplication variant, and eight were missense variants. Six variants in twelve cases were associated with epilepsy (Anazi et al., 2017; Barel et al., 2017; Sagie et al., 2018; Mitani et al., 2021), including the present case. Among the 12 patients, there were 7 males and 5 females. We identified five *TRAK1* homozygous variants (c.287-2A > C, c.287-2A > G, c.1759dupC, p.His587Profs*32; c.350G > A, p.Arg117Gln; and c.986T > C, p.Leu329Pro) in eleven patients, six of whom carried homozygous truncating variants (c.287-2A > C), including three males and three females. Three patients carried c.287-2A > G, p.His587Profs*32, and p.Arg117Gln variants. Two patients carried the variant p.Leu329Pro. We identified the novel compound heterozygous variants p.Arg279Cys and p.Lys854Gln. Figure 3 presents the genomic location of all *TRAK1* variants, a schematic diagram of the *TRAK1* protein, and the locations of variants associated with epilepsy. All patients displayed developmental delays of varying degrees. Most patients presented with infancy- or childhood-onset seizures (1–19 months). Eight patients (8/12, 67%) had epileptic spasms, and seven (7/12, 58%) were intractable to ASMs. Eight patients experienced refractory status epilepticus, of which seven (88%) died in early life. All variants showed a classical autosomal recessive inheritance pattern, and patients carrying biallelic *TRAK1* variants (including homozygous or compound heterozygous variants) had epilepsy with developmental disorders. The clinical details of the 12 patients with epilepsy are presented in Table 2.

**TABLE 2:** Patients with *TRAK1* variants with epilepsy.

Reference	Age at examination/sex	Country	Variant (NM_001042646)	Zygosity	Age at onset	Epilepsy/ seizure type	Other clinical symptoms	Brain MRI/CT	EEG	Effective ASMs and outcome of epilepsy
Barel O	NA/M	Arab	287-2A > C	Homozygous	19 mons	Myoclonic jerks, tonic-clonic seizure, spasticity, SE	Moderate delay, alert, loss of all development milestones	Numerous bilateral subcortical white matter foci, generalized atrophy	Multiple independent spike foci	Resistant to all ASMs, died at 30 mons
Barel O	NA/F	Arab	287-2A > C	Homozygous	14 mons	Generalized tonic-clonic seizure, continuous polymyoclonus, spasticity, SE	Alert, severe hypotonia and weakness, decreased alertness, respiratory distress, failure to thrive, mild delay	Generalized atrophy	Multifocal polyspike wave activity	Resistant to valproate and clonazepam, died at 18 mons
Barel O	NA/M	Arab	287-2A > C	Homozygous	2.5 mons	Myoclonic jerks, spasticity, SE	Moderate DD	Mild frontal atrophy	Considered normal	Unresponsive to various ASMs, died at 40 mons
Barel O	NA/M	Arab	287-2A > C	Homozygous	13 mons	Myoclonic jerks, spasticity, SE	Mild DD	Mild progressive cortical atrophy	Occasional bilateral independent spikes	Responded to combined ASMs, died at 17 mons
Barel O	NA/F	Arab	287-2A > C	Homozygous	1.5 mons	Myoclonic jerks, polymyoclonus, spasticity, SE	Moderate DD	NA	Normal interictal EEG	Intractable to various ASMs, died at 60 mons
Barel O	NA/F	Arab	287-2A > C	Homozygous	1 month	Myoclonic jerks, spasticity, SE	Alert, lost most of developmental milestones	Considered normal	Normal interictal EEG	Unresponsive to ASMs, alive
Anazi S	6 mons/M	NA	c.287-2A > G	Homozygous	NA	Spasticity	Global DD, microcephaly, central hypotonia, failure to thrive	Brain atrophy	NA	NA
Anazi S	7 mons/M	NA	c.1759dupC	Homozygous	NA	NA	Neonatal respiratory distress, abnormal facial shape, DD	Brain atrophy	NA	NA
Mitani T	15 mons/F	Turkish	c.350G > A	Homozygous	NA	NA	Moderate DD, hypotonia, dysmorphic features	Corpus callosum dysgenesis	NA	NA
Sagie S	NA/M	Ashkenazi-Jewish	c.986T > C	Homozygous	12 mons	Tonic-clonic, SE	Hyperekplexia, mildly delayed	NA	NA	Unresponsive to ASMs, died following SE after 12 days
Sagie S	NA/F	Ashkenazi-Jewish	c.986T > C	Homozygous	18 mons	Tonic-clonic, SE	Hyperekplexia, mildly delayed	Hyperintense lesion in the right thalamus, bilateral tissue loss in the Sylvian fissure	NA	Unresponsive to ASMs, died following SE after 17 days
Present study	13 mons/M	China	c.835C > T, c.2560A > C	Compound heterozygous	5 mons	Spasticity	Mildly delayed	Sylvian fissure dilation	Atypical hypsarrhythmia and spasms discharge	Valproate and ACTH, seizure free

ACTH, adrenocorticotropic hormone; ASMs, anti-seizure medicines; CT, computed tomography; DD, developmental delayed; EEG, electroencephalogram; F, female; M, male; mons, months; MRI, magnetic resonance imaging; NA, not available; SE, status epilepticus.

## 4 Discussion

The *TRAK1* gene, also known as *OIP106*, contains 16 exons on chromosome 3p22.1. *TRAK1* is widely expressed in the spinal cord and specific brain regions. *TRAK1* is a key regulator of mitochondrial movement and regulates mitochondrial fusion-fission and endocytic GABA-A receptor trafficking (Barel et al., 2017). In the present study, biallelic *TRAK1* missense variants were identified in a pediatric patient with epileptic spasms and developmental disorders. The two variants had no or low frequencies in the gnomAD database, are highly conserved in animals, and were predicted to be damaging by at least two *in silico* tools. Compound heterozygous pairs contained at least one variant with changes in hydrogen bonding or protein stability. These data suggest that *TRAK1* is expressed in the brain and is essential for neuronal cell function. The findings further show that compound heterozygous *TRAK1* variants are associated with epilepsy.

Previously, genome-wide linkage analysis showed that *TRAK1* is associated with childhood absence epilepsy (Chioza et al., 2009). Recessive *TRAK1* variants have been associated with DEE68 (Barel et al., 2017), which is characterized by neurodevelopmental delay, seizures, and fatal encephalopathy. *TRAK1* variants were mainly associated with epileptic seizures presenting as myoclonic and/or spastic jerks. Most brain MRI and computed tomography analyses showed abnormalities such as cortical atrophy, and the EEG showed epileptiform discharges. In the present case, the patient showed early-onset epileptic spasm seizures with developmental delay, Sylvian fissure dilation on brain MRI, bilateral posterior atypical hypsarrhythmia, and epileptic spasms in clusters based on EEG. Trio-based WES identified a novel *TRAK1* compound heterozygous variant. Gesell Developmental Observation-Revised screening revealed a mild delay. These findings indicated that the clinical manifestations were associated with epilepsy caused by the *TRAK1* variant. To the best of our knowledge, this is the first reported case of epilepsy caused by a *TRAK1* compound heterozygous variant. A meta-analysis of *TRAK1*-associated epilepsy revealed that all patients had varying degrees of developmental disorders, such as developmental delay, microcephaly, central hypotonia, and failure to thrive. Eight patients (8/12, 67%) had epileptic spasms, and seven (7/12, 58%) were intractable to anti-seizure medications. Eight patients experienced refractory status epilepticus, of which seven (88%) died in early life. These findings indicate poor outcomes for this cohort and emphasize the need for timely treatment. In addition, eight variants in eight cases were not associated with epilepsy. They each carried *TRAK1* heterozygous variants (including six missense variants, one deletion destructive variant, and one gross duplication variant) with or without developmental disorders (Xu et al., 2011; Iossifov et al., 2014; Deciphering Developmental, and Disorders, 2017; Turner et al., 2019; Bacchelli et al., 2020; Dong et al., 2020; Qiao et al., 2021).

In previous studies, most patients carrying biallelic *TRAK1* variants were either unresponsive or resistant to ASMs. Biallelic *TRAK1* variants may present disrupted GABA-A receptors. In the case study presented here, although the patient’s seizures were not responsive to valproate, he became seizure-free after treatment with valproate and ACTH. Valproate is a broad-spectrum anti-seizure medicine that exerts antiepileptic effects by increasing GABA levels in the brain and inhibiting high-frequency neuronal activity via voltage-gated calcium, sodium, and potassium channels (Van den Berg et al., 1993; Safdar and Ismail, 2023). ACTH protects neurons and exerts antiepileptic effects by regulating the function of the hypothalamic–pituitary–adrenal axis, acting on melanocortin receptors, promoting neurosteroid production, and inhibiting nervous system inflammation (Brunson et al., 2001; Herman et al., 2016; Riikonen, 2023).

The C-terminal biallelic truncated variant of *TRAK1* interfered with intracellular trafficking of the GABA-A receptor in mice (Gilbert et al., 2006). Patient cells had very little or no detectable expression of the mutant transcripts, consistent with nonsense-mediated mRNA decay, as well as severely reduced TRAK1 protein expression. This suggests an underlying mechanism for *TRAK1* loss of function. Patient cells showed irregular patterns of mitochondrial scattering with abnormal subcellular localization, decreased mitochondrial motility, reduced mitochondrial membrane potential, and decreased oxygen consumption and respiratory capacity compared to controls. The proper distribution of mitochondria in neurons and their axons is essential for high energy and calcium buffering during synaptic neurotransmission (Barel et al., 2017). *TRAK1* deficiency affects mitochondrial localization and may further influence local adenosine triphosphate (ATP) levels and ATP-dependent synaptic functions (Wu et al., 2021). Notably, mitochondrial abnormalities have been associated with epilepsy (Chan et al., 2019). *In vitro* and *in vivo* studies revealed that *TRAK1* expression was downregulated in the temporal lobe and hippocampus of patients. Knockdown of *TRAK1* in CA1 neurons shortened the time from the first episode to status epilepticus and increased the frequency of epileptic seizures (Wu et al., 2021). These findings suggest that changes in *TRAK1* expression may be associated with epilepsy pathogenesis.

The diploid human genome likely has more recessive variants than dominant ones, as indicated by the nearly double recessive inheritance genes (1,936 recessive versus 1,008 dominant) in the OMIM database (Luo et al., 2022). In this study, patients with epilepsy carried biallelic *TRAK1* variants. Thus, more attention should be paid to the role of recessive variants in epilepsy.

A limitation of this study is that the direct functional effects of the variants were not examined based on analyses of patient cells in laboratory tests.

## 5 Conclusion

We identified a novel, rare compound heterozygous *TRAK1* variant in a pediatric patient with epilepsy and a mild developmental disorder. The two variants are highly conserved in animals and predicted to be damaging. We identified biallelic *TRAK1* variants in patients with epilepsy and developmental disorders, who experienced seizures that progressed to status epilepticus. Our study highlights the diagnostic and prognostic importance of detecting *TRAK1* variant expression in patients with epilepsy. Our study emphasizes the diagnostic and prognostic importance of detecting *TRAK1* variant expression in patients with epilepsy.

## Data availability statement

The datasets presented in this study can be found in online repositories. The names of the repository/repositories and accession number(s) can be found below: NCBI GenBank: OR863198 and OR863199.

## Ethics statement

This study adhered to the guidelines of the International Committee of Medical Journal Editors on patient consent for research or participation and was approved by the Ethics Committee of the Affiliated Hospital of Zunyi Medical University. Written informed consent for participation in this study was provided by the participants’ legal guardians.

## Author contributions

R-KL: Writing – original draft. Y-RX: Writing – original draft. S-JP: Data curation, Writing – original draft. W-TL: Formal Analysis, Writing – original draft. X-MS: Project administration, Software, Writing – original draft. X-QS: Methodology, Resources, Writing – original draft. M-QT: Writing – review & editing, Writing – original draft.

## Footnotes

1
https://www.informatics.jax.org/allele/MGI:3044768

2
https://gnomad.broadinstitute.org/

3
http://www.genebee.msu.su/spdbv/text/getpc.htm

4
http://www.genemed.tech/varcards/

5
https://folding.biofold.org/i-mutant/
